# Pulmonary Embolism in Pneumonia: Still a Diagnostic Challenge? Results of a Case-Control Study in 100 Patients

**DOI:** 10.1155/2016/8682506

**Published:** 2016-05-25

**Authors:** Maria Paparoupa, Loukia Spineli, Theodor Framke, Huy Ho, Frank Schuppert, Adrian Gillissen

**Affiliations:** ^1^Intensive Care Unit, University Hospital of Giessen, 35392 Giessen, Germany; ^2^Institute of Biostatistics, Hannover Medical School (MHH), 30625 Hannover, Germany; ^3^Department of Gastroenterology, Endocrinology, Diabetology and General Medicine, Klinikum Kassel, 34125 Kassel, Germany; ^4^Department of Pulmonary Medicine, Klinikum Kassel, 34125 Kassel, Germany

## Abstract

This study evaluated the diagnostic value of D-dimer, CRP, and leucocytes count to detect an underlying pulmonary embolism (PE) in patients with pneumonia. A predictive model of an underlying PE, based on laboratory markers and clinical symptoms, was our ultimate objective. Overall 100 patients underwent a computed tomography angiography (CTA) of the lung: 54 with coexistence of PE and pneumonia (cases) and 46 with pneumonia without PE (controls). Cases and controls were matched 1 : 1. Symptoms and paraclinical findings were registered on admission. Receiver operating characteristic (ROC) curves, search for an optimal threshold, and conditional logistic regression analysis were conducted. D-dimer has a moderate ability to detect PE in pneumonia. Sensitivity of D-dimer was estimated at 97.78% and specificity at 11.11%. No optimal cut-point has acceptable diagnostic ability. After excluding patients with sepsis, sensitivity was reduced to 96.97%, whereas specificity increased to 16.13%. Consolidation in chest X-ray and positive D-dimer predict better an underlying PE as D-dimer itself. Thus, discriminatory power of the prediction model (AUC of 0.740) is not much greater than D-dimer (AUC of 0.703). No threshold that could increase the diagnostic value of D-dimer or a prediction model which is significantly better than D-dimer itself was identified.

## 1. Introduction

 The coexistence of pulmonary embolism (PE) and pneumonia (known as infarction pneumonia) is a common diagnosis in the setting of emergency medicine worldwide. The differential diagnosis towards community acquired pneumonia (CAP) remains difficult and requires the performance of a computed tomography pulmonary angiography (CTPA). The term “pneumonia” is used to describe both subtypes of pneumonia that are compared in this paper: pneumonia after pulmonary embolism which is mentioned as “infarction pneumonia” and pneumonia caused by bacterial or viral pathogens without pulmonary infarction, which is mentioned as “community acquired pneumonia.”

The existence of an underlying PE when pneumonia is diagnosed is of a highly therapeutic importance, as in this case initiation of anticoagulation treatment is required. Moreover, the computed angiography of the lung is an expensive and possibly harmful method, due to radiation and iodine exposure, which may lead to serious adverse events. Therefore, a prognostic model of an underlying PE, based only on patient's symptoms and paraclinical findings on admission, could be extremely useful in order to select cases with a high PE possibility which should undergo CTPA, in order to confirm or exclude the diagnosis. This clinical practice could contribute to the reduction of underdiagnosed cases. On the other hand, unnecessary performance of a computed tomography can be avoided in patients with low pulmonary embolism profile.

D-dimer is a laboratory biomarker that may be a candidate diagnostic test for the diagnosis of PE, since it has been used for the detection of thrombus. The cut-offs for D-dimer concentration are well known for an underlying PE without pneumonia [1]. However, the cut-offs for this parameter are unknown when pulmonary embolism coexists with pneumonia. It should be noted that D-dimer concentration increases considerably in patients with sepsis and, hence, its ability to successfully detect PE is getting dubious.

## 2. Objectives of the Study

The aim of this biomarker Phase II study is to perform a series of evaluations regarding the diagnostic value of easily and fast provided laboratory parameters such as D-dimers, CRP, and leucocytes count to successfully detect an underlying PE in patients with pneumonia. In addition, the diagnostic value of D-dimer as a marker of an underlying PE will be reevaluated after excluding patients with sepsis (sensitivity analysis). Clinical symptoms, such as fever, chest pain, syncope, dyspnea, hemoptysis, and cough, as well as echocardiographic and common X-ray findings will be also assessed between patients with PE and infarction pneumonia and patients with CAP. A predictive model of an underlying PE in patients with pneumonia is the ultimate objective of this study.

## 3. Study Design and Patients

In order to predict an underlying PE in patients with pneumonia, we designed a retrospective case-control study between subjects with coexistence of PE and infarction pneumonia and subjects with community acquired pneumonia (CAP) without pulmonary infarction. We examined the initial sample size of 600 medical records of patients diagnosed with pneumonia between 2012 and 2014 in the general hospital of Klinikum Kassel, Germany. The diagnosis of pneumonia was made according to radiological criteria, after chest X-ray, computed angiography of the lung, or both. Only 100 individuals had undergone a computed tomography angiography (CTA) of the lung; 54 were diagnosed with coexistence of PE and infarction pneumonia and 46 were diagnosed with pneumonia without PE (CAP). We selected exclusively these patients for the next step because an underlying PE can only be confirmed or excluded after a CTPA. Subsequently, patients with coexistence of PE and infarction pneumonia (cases) were matched at 1 : 1 ratio with CAP (controls), according to gender and age, in order to prevent confounding issues. Matching was not achieved for 9 cases and 1 control, leading to their exclusion from the data analysis. None of the selected patients had a former history of pulmonary embolism or an oral vitamin-K antagonist medication for any indication.

For each patient major and minor symptoms on admission were registered: fever and chest pain were referred to as major symptoms, whereas dyspnea, cough, dyspnea and cough, hemoptysis, and syncope were referred to as minor symptoms. The categorisation of patients according to their gender, age, and admission symptoms is demonstrated in Table 1.

Laboratory parameters such as C-reactive protein (CRP), total leucocytes count, and D-dimers, as well as the existence of consolidation in chest X-ray, were identified on admission. The D-Dimer Assay used for laboratory testing was “D-Dimere Test Innovance” from Siemens. Only 6 cases underwent directly a CTPA without a conventional chest X-ray first (because of the high clinical suspicion of PE). 28 cases fulfilled the criteria of sepsis (SIRS—Systemic Inflammatory Distress Syndrome—and at least one organ failure). Finally, the extension of the PE (central or subsegmental) and echocardiographic findings of a right ventricular hypertension (RVH) were collected only in PE cases. Comorbidities classically combined with RVH were registered using the medical history of each patient (see Tables 2 and 3).

## 4. Statistical Analysis

Baseline characteristics of the matched cases-controls were analyzed descriptively and compared with paired *t*-tests for quantitative data whereas McNemar tests and Cohen's Kappa were used for binary and categorical data, respectively, to assess the agreement between the two groups.

### 4.1. Evaluation of the Diagnostic Value

Receiver operating characteristic (ROC) curves were used for the evaluation of the diagnostic value of the laboratory parameters D-dimer, CRP, and leucocytes count. To interpret the area under the ROC curves (AUC), the traditional academic point system was used; 0.50–0.60 denotes very low diagnostic performance of the test, 0.60–0.70 low, 0.70–0.80 moderate, 0.80–0.90 good, and 0.90–1 excellent performance [2]. The laboratory parameter with at least a moderate ability to discriminate pneumonic patients with underlying PE was selected for the subsequent analysis.

To assess the diagnostic accuracy of the selected laboratory parameters sensitivity and specificity, as well as positive predictive value (PPV) and negative predictive value (NPV), were calculated using a 2 × 2 contingency table at a cut-point of 0.55. Accuracy was also calculated, as it reflects the probability of a correct test result and the odds ratio (OR) as another useful measure of diagnostic accuracy. Note that all these aforementioned measures were recalculated after excluding patients with sepsis.

### 4.2. Graphical Analysis for the Optimal Threshold

Optimal thresholds were detected visually using a plot proposed by Harris [3] that illustrates both sensitivity and specificity curves for all possible cut-offs of the selected laboratory parameter. In particular, the coordinates of the intersection of sensitivity with specificity reflect the probability value of sensitivity-specificity equality and the candidate optimal threshold of the studied test.

### 4.3. Conditional Logistic Regression Analysis for the Prediction Model

Among laboratory parameters evaluated, only those with at least moderate ability to discriminate pneumonic patients with underlying PE were considered in the prediction model. Clinical symptoms were evaluated regarding their association with the coexistent PE status. In the light of matched cases-controls, the appropriate analysis is conditional logistic regression that provides conditional estimates of the OR [4]. Specifically, multiple conditional logistic regression models were implemented with dependent variable the probability of “coexistent PE and pneumonia” and independent variables of at least one clinical symptom and the selected laboratory parameter, which was maintained in all studied models. The selection of the best fitted model was based on Akaike's (AIC) criterion; the smaller the AIC value is the better the model fits the data. Then we retrieved the prediction probabilities of the model and used them as the “new diagnostic test.” Ultimately, we compared the ROC of the prediction model with the ROC of D-dimer itself.

### 4.4. Secondary Analysis

As a secondary analysis we assessed the impact of embolism extension and the development of RVH on D-dimer concentration. A multivariable regression model was employed where D-dimer concentration was the dependent variable, whereas the extension of embolism, the development of RVH, and their interaction were the independent variables.

In all model-based analysis significance was inferred at a level of 0.05. Please note that significance should not be interpreted in a strict confirmatory sense. All statistical analyses were conducted using SAS 9.3.

## 5. Results

### 5.1. Demographic and Baseline Characteristics

Matching was successful for 45 pairs of patients with age difference of 2 years on average and a range from 0 up to 7 years. As shown in Table 1, gender was similarly distributed in both groups. Patients were on average age of 66 years old and the majority was male (around 60%). Fever status was similarly distributed in both groups and was present in approximately one-third of the study population. Chest pain was present mostly in patients with PE and pneumonia (53%) and it was absent in 64% of controls. Regarding the category “other symptoms,” dyspnea, syncope, and no other symptoms were more present in the PE group, whereas hemoptysis and combination of dyspnea and cough was predominant in the CAP group.

More than a half had an X-ray consolidation in both study groups. However, information on consolidation status could not be retrieved in 6 cases, as these ones underwent directly a CTPA. Approximately one-third of the study subjects in both groups fulfilled the sepsis criteria (SIRS and at least one organ failure). The average D-dimer value was at least 2 times higher in patients with coexistent PE and pneumonia than in controls and this difference was significant based on paired *t*-test (mean difference 4.04, *p* value 0.003). Furthermore, patients with coexistent PE and infarction pneumonia had on average lower CRP values and leucocyte counts than controls, but these differences were not statistically significant (*p* value 0.695 and 0.650, resp.). Among patients with PE, approximately 47% had a central embolism and 60% had no echocardiographic signs of RVH. Most interestingly, the majority (55.55%) of patients with right ventricular hypertension (RVH) had relative comorbidities; 33.33% left ventricular insufficiency (LVI); and 22.22% chronic obstructive pulmonary disease (Table 3).

### 5.2. Evaluating the Diagnostic Value of D-Dimer, CRP, and Leucocytes

**Figure 1:**
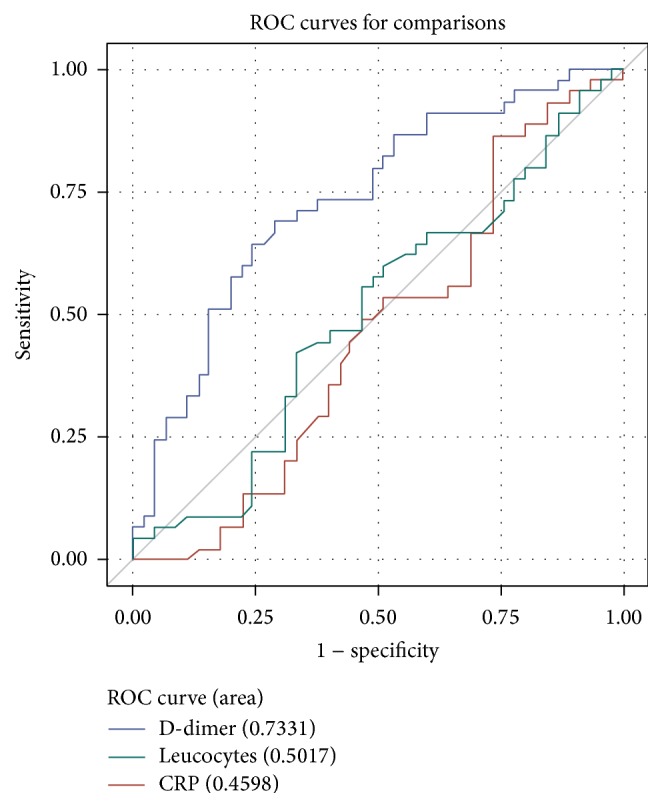
ROC curves for D-dimer, CRP, and Leucocytes.

To evaluate the diagnostic value of the laboratory parameters D-dimer, CRP, and leucocytes to successfully detect an underlying PE, we compared the receiver operating characteristic curves (ROC) of these parameters.  Figure 1 presents the ROC for each parameter using different colors along with the respective area under the curve (AUC). D-dimer has AUC of 0.733 and, hence, its discriminatory power seems to be moderate. CRP and leucocytes yield an AUC of 0.460 and 0.502, respectively; both parameters have a very poor diagnostic accuracy and are likely to diagnose patients with coexistent PE at random.

### 5.3. Evaluating the Diagnostic Value of D-Dimer Using a Known Threshold

As shown in Tables 4(a) and 4(b), D-dimer achieves high true positive and false positive results as opposed to true negative and false negative results which are very low. Sensitivity is estimated at 97.78% and specificity at 11.11% and, therefore, D-dimer is very sensitive for the detection of an underlying PE in the setting of pneumonia, without being specific. PPV indicates that 52% with a positive test result actually have a PE, whereas the remaining high proportion of 48% estimated to have PE does not. Furthermore, according to NPV, 83% of patients with negative test result do not have coexistent PE and, consequently, the risk of overlooking a PE is actually low. The accuracy of D-dimer was 54.44%.

### 5.4. Searching for the Optimal D-Dimer Threshold

**Figure 2:**
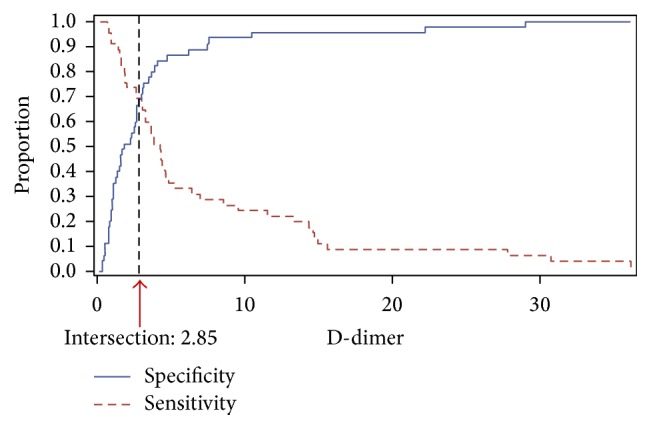
Plot by Harris for sensitivity and specificity of D-dimer across all cut-off values.

Using the plot proposed by Harris, sensitivity and specificity intersect at the value of 2.85 mg/L FEU which corresponds to sensitivity and specificity equal to 68.89% (Figure 2). Compared to the cut-off at 0.55 mg/L FEU used in the study, sensitivity decreased by 36%, whereas specificity increased by 520%. Consequently, D-dimer impairs its ability to correctly detect patients with PE but gained its ability to correctly detect patients without PE. Sensitivity decreases whereas specificity increases with a cut-off value above 2.85 mg/L FEU. In contrast, sensitivity increases whereas specificity decreases for cut-off values below 2.85 mg/L FEU. To achieve a sensitivity of at least 90%, the best cut-point could be 0.95 mg/L FEU, with sensitivity 91.1% and specificity 24.4%. Using the unweighted Youden index, the optimal cut-point is 0.4 mg/L FEU with corresponding sensitivity of 100% and specificity of 6.7%. Taking into account these results we conclude that* no optimal cut-point for D-dimer seems to reach acceptable diagnostic ability*. Excluding the patients with sepsis reduces the sample to 64 patients. Sensitivity is reduced negligibly to 96.97%, whereas specificity increased to 16.13%, which still renders as low D-dimer's specificity ability.

### 5.5. Prediction Model of an Underlying PE in Patients with Pneumonia

The regression coefficients for the covariates D-dimer and symptoms were interpreted in the exponential scale, that is, as odds ratios.  Table 5 presents the studied covariates along with their levels and the coded values we used in the models. Results on AIC from the multiple conditional logistic regression models are presented in Table 6. According to AIC values, illustrated in Table 6, the model with fever (model 5) seems to fit worst the data because it yields larger AIC value (AIC 55.33) than the model including only D-dimer (model 1; AIC 54.15), whereas the model including only “consolidation” (model 4) seems to fit better the data, since it has the smallest AIC value (AIC 51.54). Therefore, model 4 will be the prediction model of an underlying PE in patients with pneumonia.

### 5.6. Comparing the Prediction Model with D-Dimer in Terms of Discriminatory Power

**Figure 3:**
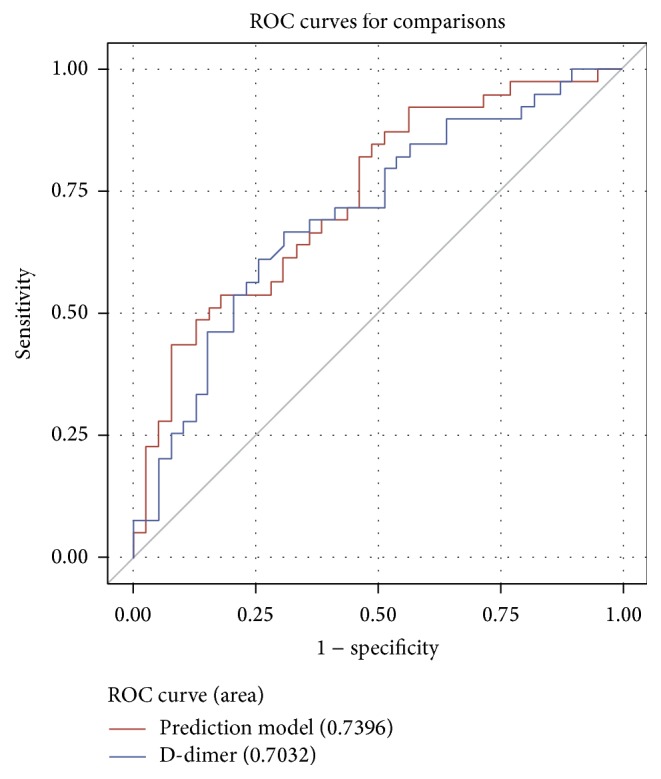
ROC curves for D-dimers and prediction model.

As shown in Figure 3, D-dimer has AUC of 0.703 which reflects low discriminatory power, whereas the prediction model has an AUC of 0.740. Both parameters reflect quite similar moderate discriminatory power with a small advantage of the prediction model.

**Figure 4:**
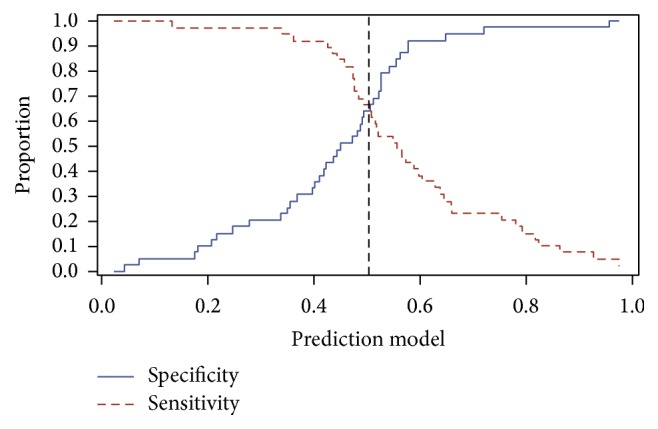
Line plot of sensitivity and specificity under all possible cut-offs of the prediction model.

Since the prediction model seems to have a little bit better discriminatory power than D-dimer, the last step is to search for the optimal cut-point of the prediction model. Using again the plot by Harris (Figure 4), sensitivity and specificity intersect at the value of 0.50 which corresponds to sensitivity and specificity equal to 64.1%. In order to achieve sensitivity and specificity at least 90%, two cut-points were chosen arbitrarily: 0.30 mg/L FEU with sensitivity 97.4% and specificity 23.1% and 0.80 mg/L FEU with sensitivity 12.8% and specificity 97.4%. Using the unweighted Youden index, the optimal cut-point is 0.36 mg/L FEU with corresponding sensitivity of 92.3% and specificity of 28.2%.

### 5.7. Correlation between D-Dimer and Extension of PE and Right Ventricular Hypertension

To answer the questions (i) whether patients with a central embolism are expected to have higher D-dimer than those with subsegmental embolism and (ii) whether patients with a RVH are expected to have higher D-dimer than those without, we implemented a multivariable regression model with D-dimer concentration as dependent variable and the variables embolism (“subsegmental” as reference level) and RVH (“no” as reference level) and their interaction as independent variables.

**Figure 5:**
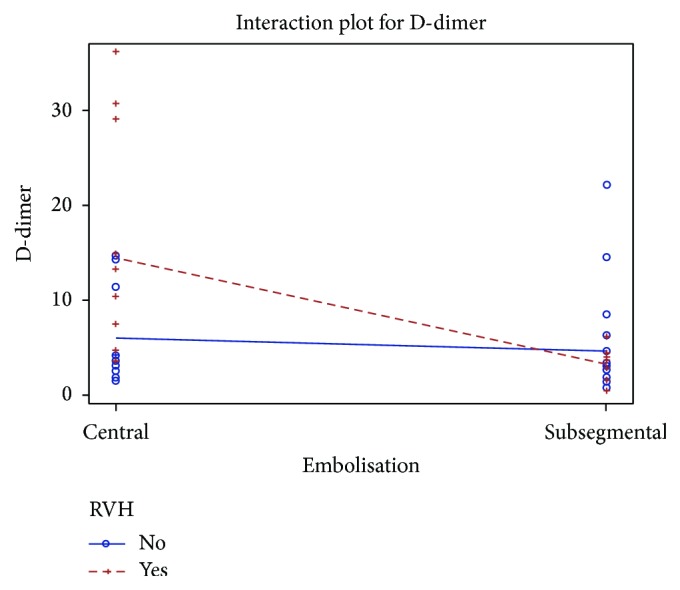
Correlation between D-dimer and extension of PE and right ventricular hypertension.

As shown in Figure 5, in the absence of RVH, D-dimer is on average around 6.24 mg/L FEU in patients with central and 4.67 mg/L FEU in those with subsegmental pulmonary embolism, but in the presence of RVH, D-dimer is on average around 14.45 mg/L FEU in patients with central as opposed to around 3.38 mg/L FEU in those with subsegmental embolism. Therefore, RVH correlates with D-dimers in patients with central pulmonary embolism but not in those with subsegmental one.

## 6. Discussion

The diagnosis of pneumonia in emergency setting is based on patient's symptoms, laboratory tests, and chest X-ray consolidation, but the origin remains unclear (infarction pneumonia as a result of pulmonary embolism or community acquired pneumonia caused by infectious pathogens without underlying pulmonary infarction) as long as no CTA is performed. Despite that, not all patients diagnosed with pneumonia can or should undergo a CTA. Therefore, our purpose was to create a model based on parameters that can be easily collected by admission in order to predict which patients with pneumonia are at a high risk for an underlying pulmonary embolism and therefore should undergo a CT angiography of the lung to get the diagnosis confirmed or excluded. We conducted a retrospective study including only subjects where CTA was already performed because this design allows the comparison between the two groups. However, a prospective study could be feasible after having a prediction model as a decision making tool. We collected clinical and paraclinical characteristics on admission and tested them for their diagnostic accuracy separately and in combination.

D-dimer performs moderately but significantly better than CRP and leucocytes count in order to predict an underlying PE in patients with pneumonia. The exclusion of septicemic patients, considering that D-dimer increase in sepsis, brought only a slight increase of the diagnostic accuracy of the marker. The selected threshold of 0.55 mg/L FEU provides a high sensitivity but a low specificity. This information is already known in the setting of pulmonary embolism [5] but insufficiently investigated in case of concomitant infarction pneumonia [1].

In the question for an optimal D-dimer threshold, we compared a “candidate” cut-off of 2.85 mg/L FEU where sensitivity and specificity are equal to 68.89% with the given cut-off at 0.55 mg/L FEU. According to the plot analysis proposed by Harris (REF) sensitivity decreases whereas specificity increases for cut-off value above 2.85 mg/L FEU and, in contrast, sensitivity increases whereas specificity decreases for cut-off values below 2.85 mg/L FEU. There is subsequently no optimal cut-point for D-dimer reaching an acceptable diagnostic ability. Interestingly, an age-adjusted D-dimer value (AADD) has been proposed lately, in order to increase the clinical usefulness of the marker (due to the literature, AADD is calculated as patient's age *∗* 10 ng/mL, in patients aged > 50 years) [6]. This calculation was not performable in our protocol, as we initially matched cases and controls according to gender and age.

Considering the clinical symptoms on admission, fever was almost equally present in both categories. This was an interesting finding as most of clinicians combine fever exclusively with infection. Chest pain was clearly more often in the group of PE, but no statistical difference was observed in comparison to the CAP. Shortness of breath (dyspnea) and cough, as long as hemoptysis were observed predominantly in the CAP group, and syncope in the PE group. The distribution of symptoms seems to be different in two similar studies. In the study of Zhang et al. chest pain, shortness of breath, hemoptysis, and fever were identified as independent risk factors of a PE in patients preliminarily diagnosed with pneumonia [7]. On the other hand, Söderberg et al. highlighted presence of dyspnea and/or chest pain in pulmonary embolism and fever, chills, and/or cough in community acquired pneumonia [8]. The reason for the heterogeneous symptoms' distribution between similar studies lies more likely in the study design or the symptoms' definition.

Nevertheless, the combination of clinical symptoms with D-dimer in multiple prediction models of an underlying PE did not perform better than D-dimer itself, with the exception of chest pain and X-ray consolidation. Ultimately, we decided to use the prediction model combining X-ray consolidation and D-dimer and compare it with D-dimer itself. ROC curve confirmed a similar discriminating power and plot analysis by Harris retrieved any optional cut-off which could increase the diagnostic accuracy of the prediction model.

In terms of the association between D-dimer, RVH, and extension of pulmonary embolism, we found that RVH correlates with D-dimers in patients with central pulmonary embolism but not in those with subsegmental one. Note that the precision of the results is very low due to a small sample of 45 patients and, hence, any conclusions should be drawn with caution. Moreover, RVH can be observed in many confounding conditions, more often secondary due to COPD or LVI, which practically impoverish the meaning of echocardiographic findings in identifying underlying PE in the setting of pneumonia.

## 7. Conclusions

Our study failed to find a prediction model of an underlying PE in pneumonia, based on clinical and paraclinical findings, which is significantly better than D-dimer itself. D-dimer is a marker of a moderate ability to identify PE when pneumonia is present. Therefore, we encourage all clinicians to perform a CTA of the lung, once they suspect pulmonary embolism in a patient diagnosed with pneumonia.

## Figures and Tables

**Table 1:** Categorisation of patients according to their gender, age, and admission symptoms.

Characteristic	Patient group		Total (*n* = 90)
	PE (*n* = 45)	CAP (*n* = 45)	
*Gender*			
Male	26 (58%)	26 (58%)	52 (58%)
Female	19 (42%)	19 (42%)	38 (42%)
*p* value (McNemar)	NA^1^		

*Age*			
Mean	66.58	66.22	66.40
Std	17.59	17.41	17.40
Median	71	71	71
Minimum	23	25	23
Maximum	92	99	99
*p* value (*t*-test)	0.381		

*Fever*			
Yes	13 (28.89%)	12 (26.67%)	25 (27.78%)
No	32 (71.11%)	33 (73.33%)	65 (72.22%)
*p*-value (McNemar)	0.808		

*Chest pain*			
Yes	24 (53.33%)	16 (35.56%)	40 (44.44%)
No	21 (46.67%)	29 (64.44%)	50 (55.56%)
*p*-value (McNemar)	0.032		

*Other symptoms*			
Dyspnea	18 (40.00%)	16 (35.56%)	34 (37.78%)
Dyspnea and cough	5 (11.11%)	15 (33.33%)	20 (22.22%)
Hemoptysis	0 (0.00%)	3 (6.67%)	3 (3.33%)
Syncope	6 (13.33%)	3 (6.67%)	9 (10.00%)
No other symptoms	16 (35.56%)	8 (17.78%)	24 (26.67%)
*p* value (Kappa)	0.588		

PE: pulmonary embolism; CAP: community acquired pneumonia; *n*: number of patients; Std: standard deviation; NA: not applicable; *t*-test: paired *t*-test; McNemar: McNemar test; Kappa: weighted Kappa coefficient.

^1^There are no discordant pairs.

**Table 2:** Categorisation of patients according to paraclinical characteristics.

Characteristic	Patient group		Total (*n* = 90)
	PE (*n* = 45)	CAP (*n* = 45)	
*Consolidation*			
Yes	21 (53.85%)	30 (66.67%)	51 (60.71%)
No	18 (46.15%)	15 (33.33%)	33 (39.29%)
Missing	6	0	6
*p* value (McNemar)	0.371		

*Sepsis*			
Yes	12 (26.67%)	14 (31.11%)	26 (28.89%)
No	33 (73.33%)	31 (68.89%)	64 (71.11%)
*p* value (McNemar)	0.564		

*D*-*dimer*			
Mean	7.20	3.16	5.18
Std	8.27	4.67	6.98
Median	4.10	1.85	2.77
Minimum	0.52	0.18	0.18
Maximum	36.10	27.70	36.10
*p* value (*t*-test)	0.003		

*CRP*			
Mean	98.13	107.78	102.95
Std	91.10	118.65	105.29
Median	64	64	64
Minimum	5	3	3
Maximum	404	399	404
*p* value (*t*-test)	0.695		

*Leucocytes*			
Mean	11671.11	12213.33	11942.22
Std	4425.83	6343.34	5445.29
Median	10800	11600	10950
Minimum	4100	4900	4100
Maximum	24400	42700	42700
*p* value (*t*-test)	0.650		

PE: pulmonary embolism; CAP: community acquired pneumonia; *n*: number of patients; Std: standard deviation; CPR: C-reactive protein; *t*-test: paired *t*-test; McNemar: McNemar test.

**Table 3:** Extension of PE and RVH in PE group. Comorbidities in RVH group.

Characteristic	Counts	Percentage (%)
*Embolism*		
Central	21	46.67
Subsegmental	24	53.33

*RVH*		
Yes	18	40.00
No	27	60.00

*Comorbidities RVH*		
COPD	4	22.22
LVI	6	33.33
RVH PE	8	44.44

PE: pulmonary embolism; RVH: right ventricular hypertension; COPD: chronic obstructive pulmonary disease; LVI: left ventricular insufficiency.

**(a):** 

D-dimer results on PE	CTA results on PE	
	Presence	Absence
Positive	44 (TP)	40 (FP)
Negative	1 (FN)	5 (TN)

**(b):** 

Measure	Proportion	Asymptotic 95% CI	Exact 95% CI
Sensitivity	97.78%	93.47%–100%	88.23%–99.94%
Specificity	11.11%	1.93%–20.29%	3.71%–24.05%
PPV	52.38%	41.70%–63.06%	41.19%–63.40%
NPV	83.33%	53.51%–100%	35.88%–99.58%
Accuracy	54.44%	44.16%–64.73%	43.60%–64.98%

PE: pulmonary embolism; CTA: computed tomography angiography; TP: true positive; FP: false positive; FN: false negative; TN: true negative, CI: confidence interval; PPV: positive predictive value; NPV: negative predictive value.

**Table 5:** Studied covariates along with their levels and the coded values used in the models.

Covariate	Levels	Coded value^1^
Chest pain	No	0
	Yes	1

Consolidation	No	0
	Yes	1

Fever	No	0
	Yes	1

Other symptoms	No other symptoms	0
	Dyspnea	1
	Dyspnea and cough	2
	Hemoptysis	3
	Syncope	4

^1^The levels coded with zero are the reference levels to be compared with the rest of the levels in the model.

**Table 6:** Results on AIC from the multiple conditional logistic regression models.

Model	Included variables	AIC
(1)	D-dimer	54.15
(2)	D-dimer and other symptoms	55.76
(3)	D-dimer and chest pain	53.10
(4)	D-dimer and consolidation	51.54
(5)	D-dimer and fever	55.33
(6)	D-dimer, other symptoms, and chest pain	55.69
(7)	D-dimer, other symptoms, and consolidation	53.43
(8)	D-dimer, chest pain, and consolidation	51.79
(9)	D-dimer, other symptoms, chest pain, and consolidation	54.20

AIC: Akaike information criterion.

## Additional Points

- D-dimer has a moderate ability to detect PE in the setting of pneumonia.
- No optimal cut-point for D-dimer seems to reach acceptable diagnostic ability.
- A prediction model, combining D-dimer with clinical symptoms, has a better discriminatory power than D-dimer itself, but not statistically significant.
- By clinical suspicion of a PE in a patient with pneumonia, CTPA should be conducted.
